# Mesoporous silica coated spicules for photodynamic therapy of metastatic melanoma

**DOI:** 10.1186/s12951-024-02471-y

**Published:** 2024-04-15

**Authors:** Xuejiao Liang, Jialiang Zhang, Chi Zhang, Haojie Zhai, Ping Yang, Ming Chen

**Affiliations:** 1 Department of Marine Biological Science & Technology, College of Ocean and Earth Sciences, Xiamen University, Xiamen, 361102 China; 2 State-Province Joint Engineering Laboratory of Marine Bioproducts and Technology, Xiamen University, Xiamen, 361102 China; 3 Fujian Key Laboratory of Advanced Technology for Cancer Screening and Early Diagnosis, Clinical Oncology School of Fujian Medical University, Fujian Cancer Hospital, Fuzhou, 350014 People’s Republic of China; 4 Shenzhen Research Institute of Xiamen University, Shenzhen, 518000 China; 5 Pingtan Research Institute of Xiamen University, Pingtan, 350400 China; 6 Present Address: State Key Laboratory of Pharmaceutical Biotechnology, School of Life Sciences, Nanjing University, Nanjing, 210093 China

**Keywords:** SHS, Mesoporous silica, Melanoma, Photodynamic therapy, Skin delivery

## Abstract

We report on the fabrication of mesoporous silicon dioxide coated *Haliclona* sp. spicules (mSHS) to enhance the delivery of the insoluble photosensitizer protoporphyrin IX (PpIX) into deep skin layers and mediate photodynamic therapy for metastatic melanoma in mice. The mSHS are dispersed sharp edged and rod-like micro-particles with a length of approximate 143.6 ± 6.4 μm and a specific surface area of 14.9 ± 3.4 m^2^/g. The mSHS can be topically applied to the skin, adapting to any desired skin area and lesion site. The insoluble PpIX were incorporated into the mesoporous silica coating layers of mSHS (mSHS@PpIX) with the maximum PpIX loading capacity of 120.3 ± 3.8 μg/mg. The mSHS@PpIX significantly enhanced the deposition of PpIX in the viable epidermis (5.1 ± 0.4 μg/cm^2^) and in the dermis (0.5 ± 0.2 μg/cm^2^), which was 154 ± 11-fold and 22 ± tenfold higher than those achieved by SHS, respectively. Topical delivery of PpIX using mSHS (mSHS@PpIX) completely eradicated the primary melanoma in mice in 10 days without recurrence or metastasis over 60 days. These results demonstrate that mSHS can be a promising topical drug delivery platform for the treatment of diverse cutaneous diseases, such as metastatic melanoma.

**Graphic Abstract:**

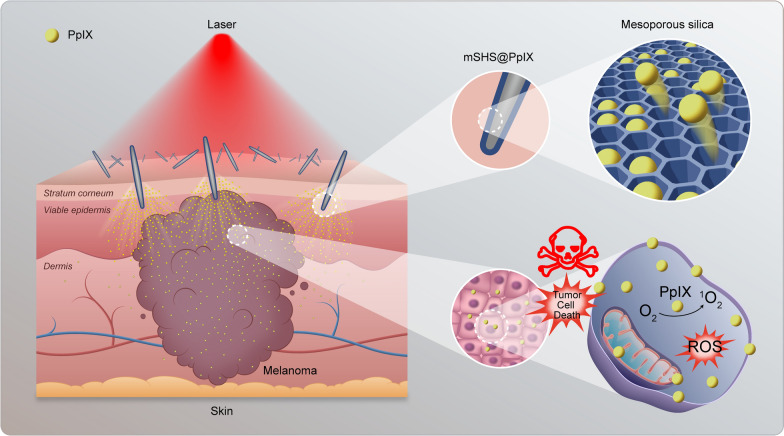

**Supplementary Information:**

The online version contains supplementary material available at 10.1186/s12951-024-02471-y.

## Highlights

- Novel Delivery Platform: mSHS offers a unique platform for topical drug delivery.
- Enhanced Penetration: mSHS@PpIX improves PpIX penetration into deep skin layers.
- Superior Deposition: mSHS@PpIX achieves higher PpIX deposition in skin compared to conventional methods.
- Effective Melanoma Treatment: Topical mSHS@PpIX eradicates melanoma in mice with no recurrence or metastasis.

**Supplementary Information:**

The online version contains supplementary material available at 10.1186/s12951-024-02471-y.

## Introduction

Melanoma is a type of malignant skin tumor originated from melanocytes due to its genetic changes or non-repaired DNA damage [1]. Metastatic melanoma (MM) is highly invasive and fatal though it only represents 4% of skin cancer incidence [2, 3]. Various strategies have been utilized for MM treatment, including surgical resection [4–6], chemotherapy [7, 8], radiotherapy [9–11], immunotherapy [12, 13], and their combinations [14, 15]. The therapeutic effects of traditional MM treatments are not ideal, primarily because they are mostly immuno-suppressive [16]. Surgical resection can impair the immunologic functions of lymphocytes and natural killer cell, which in turn leads to the spread or metastasize of tumor cells [17]. Chemotherapy and radiotherapy generally can result in significant toxicity to the bone marrow, which is the main source of immune cells [18].

Photodynamic therapy (PDT) has been widely used in the treatment of malignant tumors [17]. The key component of PDT is photosensitizer (PS), which can be activated by a specific laser to generate highly reactive singlet oxygen (^1^O_2_). These singlet oxygen molecules can further destroy cancer cells, damage tumor blood vessels, and induce apoptosis and necrosis of tumor tissue [2, 19–21]. Additionally, PDT can promote tumor tissue to release chemokines, pro-inflammatory factors and tumor antigens from tumor tissue and induce the body to produce an immune response [18]. For the treatment of MM, skin delivery of PS can bring forth many advantages over its systemic delivery (intravenous injection or oral administration), including direct access to the lesion site, reduction of the PS dosage, avoidance of the systemic skin phototoxicity, good patient compliance, among others [22–24]. However, the stratum corneum poses a significant barrier to the penetration of most therapeutics into deeper skin layers [25, 26]. While hydrophobic PSs, such as Temoporfin (m-THPC) or Protoporphyrin IX (PpIX), generally show greater incorporation into tumor cells with better PDT effects [27], they tend to accumulate in the stratum corneum and hardly diffuse into deep skin layers [18] even with the enhanced topical delivery using ethanol or different liposomal systems [28, 29]. The physical delivery techniques, such as iontophoresis, sonophoresis, microneedle patches, generally are unsuitable for treating large skin areas or non-flat lesion sites [30]. Therefore, delivering hydrophobic PSs into deep skin layers, regardless of the lesion area and site, for the MM treatment remains a significant challenge.

**Scheme 1.**
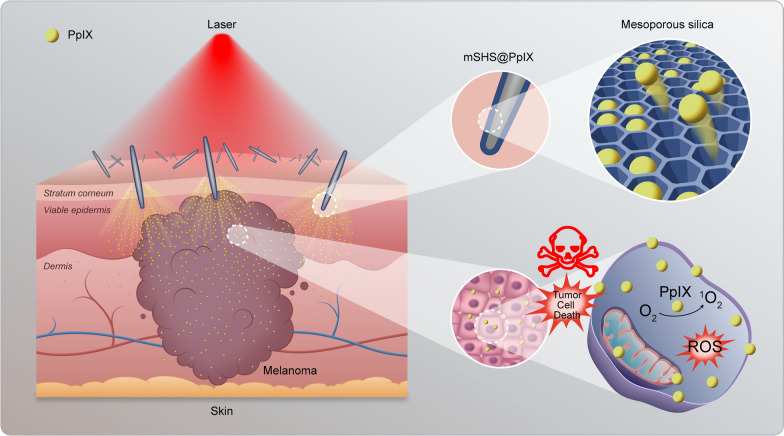
The topical application of mSHS with PpIX incorporated (mSHS@PpIX) for photodynamic therapy of metastatic melanoma. mSHS @ PpIX penetrate the skin, releasing PpIX into the skin and tumor tissue. Subsequently, PpIX generates singlet oxygen when triggered by laser irradiation (at 635 nm), which then kills tumor cells

In this study, we report on the development of mesoporous silicon dioxide-coated *Haliclona* sp. spicules (referred to as mSHS) to enhance the delivery of a hydrophobic photosensitizer, protoporphyrin IX (PpIX), into deep skin layers for the elimination of MM (Scheme 1). As discrete microneedles, mSHS can be topically applied to the skin, adapting to any desired skin area and lesion site. Furthermore, we can incorporate hydrophobic drugs into its mesoporous silica layer so that mSHS can be inserted into the skin, delivering and releasin g the loaded drugs directly into the deep skin layers.

## Results and discussion

### Fabrication and characterization of mSHS

**Fig.1:**
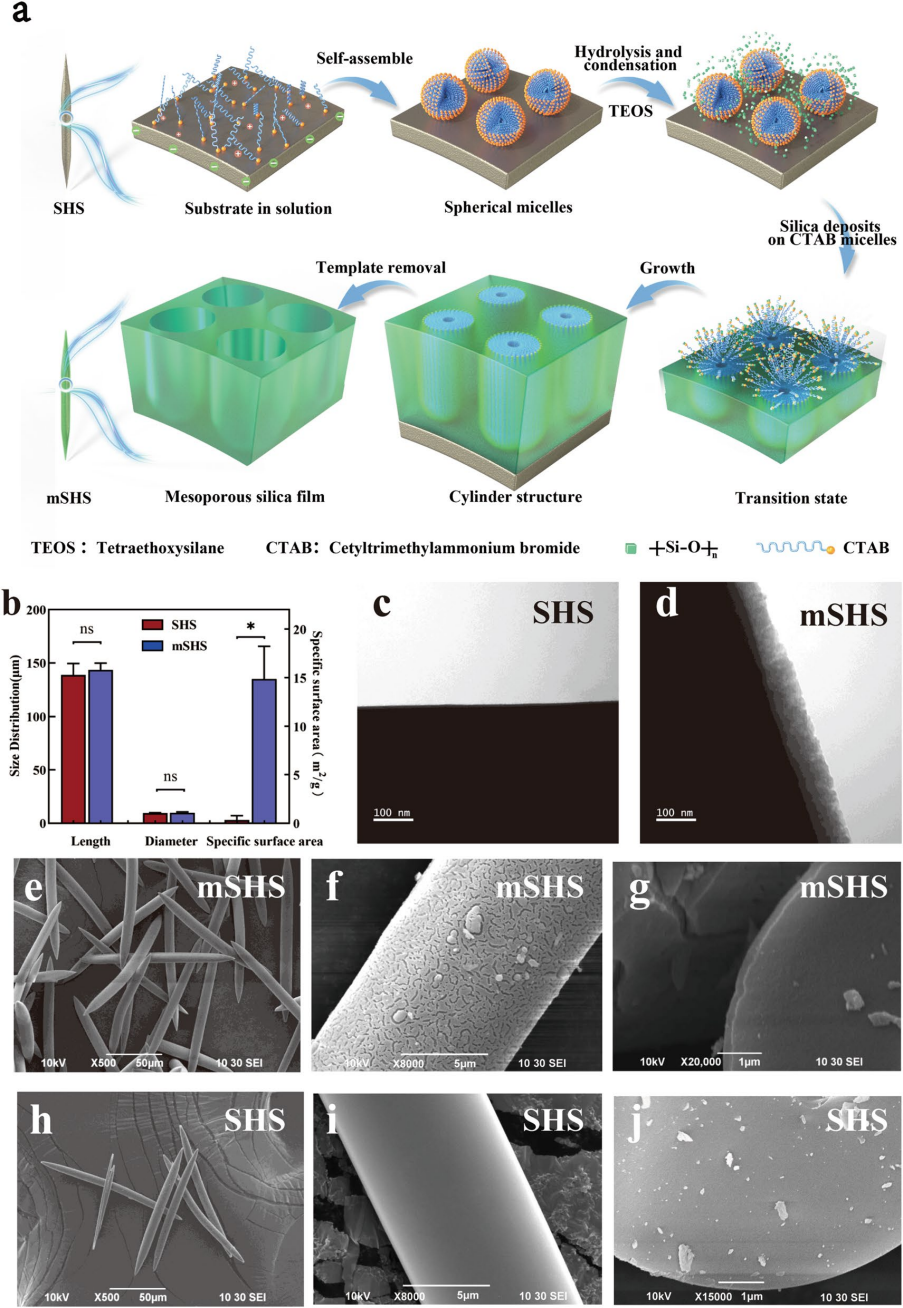
**a** Preparation schematic diagram of mesoporous silica on the surface of SHS; **b** Length, diameter and specific surface area of mSHS and SHS. The data are presented as mean ± SD (n = 3). The symbol * indicates *p* < 0.05. **c**–**j** Characterization of SHS and mSHS. **c**, **d** TEM images of SHS and mSHS; **c** TEM images of SHS, **d** TEM images of mSHS. **e**–**j** SEM images of mSHS and SHS; **e** Panorama of mSHS, **f** Partial view of mSHS surface, **g** Section of mSHS, **h** Panorama of SHS, **i** Partial view of SHS surface, **j** Section of SHS

Sponge *Haliclona* sp. spicules (SHS) are dispersed sharp edged and rod-like oxeas (Additional file 1: Fig. S1). These oxeas were prepared from cultivated sponge *Haliclon*a sp. according to our patented method (ZL201610267764.6). We fabricated mSHS by constructing a layer of mesoporous silicon dioxide on the SHS surface using the classic surfactant-templated synthesis method (Fig. 1a). While mSHS had a similar spicule size to SHS, the specific surface area of mSHS (14.9 ± 3.4 m^2^/g) was significantly higher (*p* < 0.01) than that of SHS (0.4 ± 0.3 m^2^/g) (Fig. 1b) with a specific ratio of SHS to CTAB to TEOS of 5:4:2. By observing mSHS using TEM (Fig. 1c, d) and SEM (Fig. 1e–j), we found that mSHS exhibited a characteristic appearance with a mesoporous silica coating (Fig. 1e–g), which was completely different from that of SHS (Fig. 1h–j). The thickness and pore diameter of the mesoporous silica coating layer of mSHS were 154.2 ± 7.0 nm and 9.8 ± 0.8 nm, respectively. Moreover, by regulating the concentrations of the key reaction substrates (SHS, CTAB, and TEOS), we can control the thickness of the mesoporous silica layer (Additional file 2: Fig. S2a–d). This indicates that mSHS could be utilized for delivering various drugs to the skin based on their physicochemical properties. Furthermore, we could simply physically mix the different drug-loaded mSHS (e.g., mSHS@PpIX and mSHS@FITC-dextran) (Additional file 1: Fig. S2e) for their simultaneous delivery at any desired ratio.

### Topicail delivery of PpIX into deep skin layers using mSHS

**Fig. 2:**
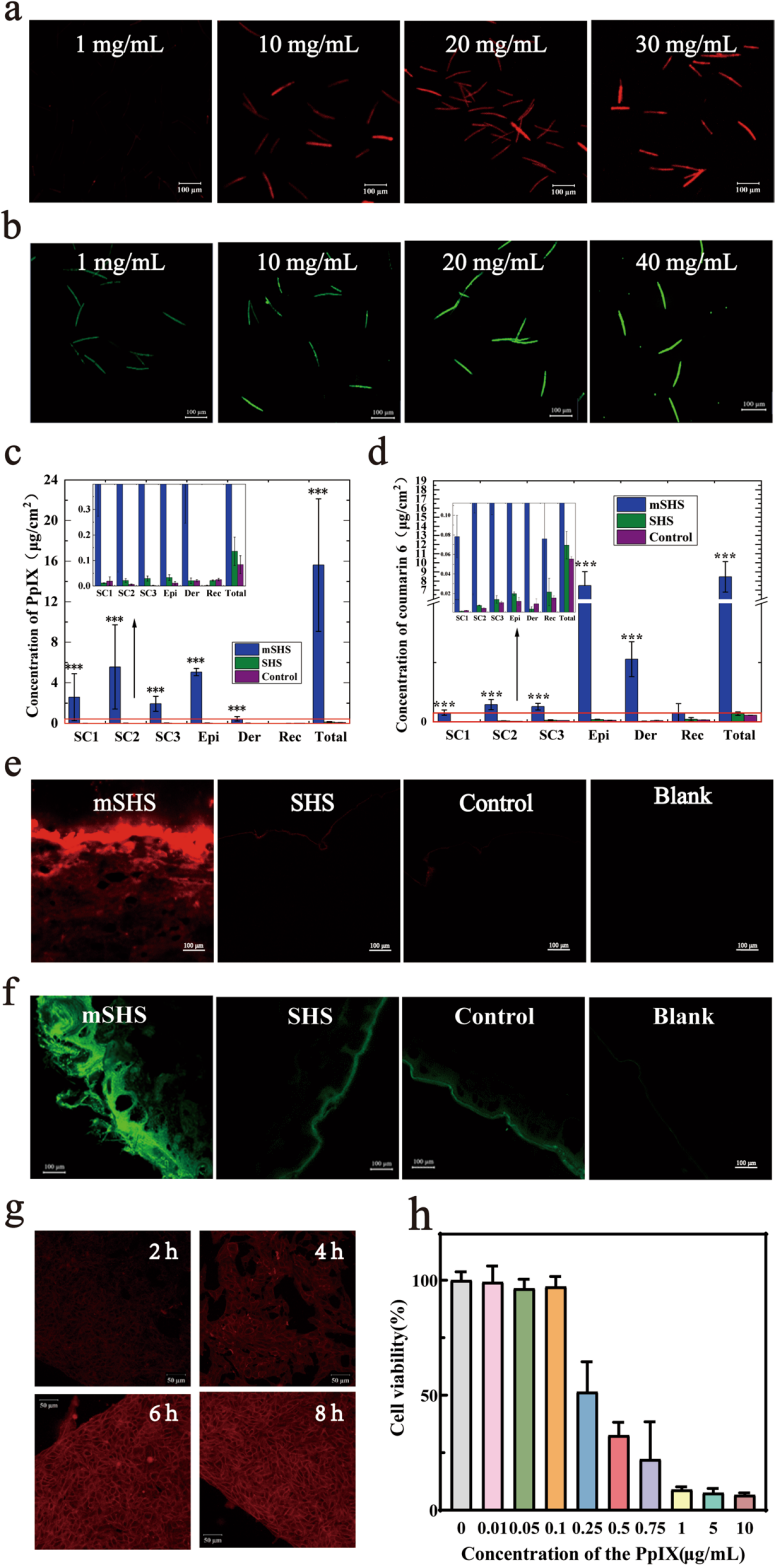
mSHS can increase the load of PpIX and coumarin 6 dramatically and enhanced skin penetration of coumarin and PpIX in vitro. **a** Visualization of mSHS@PpIX at different concentration of the PpIX. Scale bar was 100 µm. **b** Visualization of mSHS@coumarin 6 at different concentration of the coumarin 6. Scale bar was 100 µm. **c** Skin penetration of PpIX using SHS and mSHS in vitro. SC denotes 1–10 tape strip, Epi denotes the epidermis, Der denotes the dermis, and Rec denotes the receptor. ***represents *p* < 0.001. **d** Coumarin 6 penetrates different skin layer in vitro. **e** Visualization of skin delivery of PpIX. Scale bar was 100 µm **f** Visualization of skin delivery of coumarin 6. **g** The internalization of PpIX in B16 cells over time. Scale bar was 50 µm. **h** Cell viability of B16 cells at different concentrations of PpIX

We then incorporated PpIX into the mesoporous silica coating layers of mSHS (mSHS@PpIX). The amount of encapsulated PpIX in mSHS@PpIX was proportional to the concentration of PpIX in the organic solvent (Fig. 2a and Additional file 3: Fig. S3a). The maximum PpIX loading capacity of mSHS was 120.3 ± 3.8 μg/mg. Furthermore, 90.9% ± 2.3% of the PpIX loaded in mSHS was released into the PBS solution (containing 0.5% Tween80) within 24 h (Additional file 3: Fig. S3b). Similar results were obtained when using mSHS@coumarin 6 (Fig. 2b and Additional file 4: Fig. S4). It should be pointed out that mesoporous materials can also simultaneously load various drugs with different physical and chemical properties, with controlled drug release [31–35].

SHS offers a promising strategy to enhance the skin delivery of hydrophilic biomacromolecules[36] and even nanoparticles[37, 38] by effectively disrupting the skin barrier. However, SHS cannot significantly (*p* > 0.05) inrease the skin penetration of PpIX (0.14 ± 0.06 μg/cm^2^) compared to the control (saturated solution of PpIX in 30% ethanol) (0.08 ± 0.04 μg/cm^2^) (Fig. 2c). Furthermore, due to its hydrophobic nature, most of the PpIX was accumulated in the SC layers when using SHS or passive penetration. In contrast, mSHS@PpIX dramatically enhanced the skin absorption of PpIX (15.6 ± 6.5 μg/cm^2^, *p* < 0.001) compared to SHS and the control (Fig. 2c). Additionally, mSHS@PpIX significantly increased the accumulation of PpIX in deep skin layers, including viable epidermis (5.1 ± 0.4 μg/cm^2^) and dermis (0.5 ± 0.2 μg/cm^2^), which was 154 ± 11-fold and 22 ± tenfold of those (0.03 ± 0.01 μg/cm^2^ in viable epidermis and 0.02 ± 0.01 μg/cm^2^ in dermis) by SHS, respectively (Fig. 2c). These results indicate that topical application of mSHS@PpIX can not only greatly improve the drug loading of PpIX in the topical delivery system but can also directly deliver PpIX, a very hydrophobic drug, into the deeper layers of the skin. We also visualized the skin penetration and deposition behavior of PpIX in vitro using confocal microscopy (Fig. 2e). These findings have provided confirmation that the topical administration of mSHS@PpIX effectively enhances the permeation of PpIX into the deeper layers of the skin compared to other groups. Comparable findings were achieved in the in vitro transdermal administration of coumarin 6 using mSHS (Fig. 2d and f). Recently, mesoporous nanoparticles have also been used for skin drug delivery [39, 40]. However, these nanoparticles were only accumulated in the hair follicles [41, 42]. In contrast, mSHS can directly bring the mesoporous coating into deep skin layers with the drug loaded in mesoporous coating layers being released locally.

We also investigated the internalization of PpIX in B16 cells over time using confocal microscopy. PpIX was gradually accumulated in the cells in 6 h (Fig. 2g). Afterward, the fluorescence intensity of PpIX in the cells remained almost unchanged (Fig. 2g), indicating that the maximum killing rate of PpIX on B16 cells can be reached when PpIX is irradiated by laser light after at least 6 h of internalization in cells. Furthermore, We assessed the impact of varying concentrations of PpIX on the viability of B16 cells through the implementation of the MTT technique. While the cell viability of B16 cells was inversely proportional to the PpIX concentration between 0.1 μg/mL and 1 μg/mL, high concentrations (more than 1 μg/mL) of PpIX resulted in all cells dying (Fig. 2h), indicating that enhanced skin delivery of PpIX to reach its therapeutic concentration in tumor tissues is critical for the successful treatment of MM [43].

### The treatment of metastatic melanoma in mice using mSHS@PpIX

**Fig. 3:**
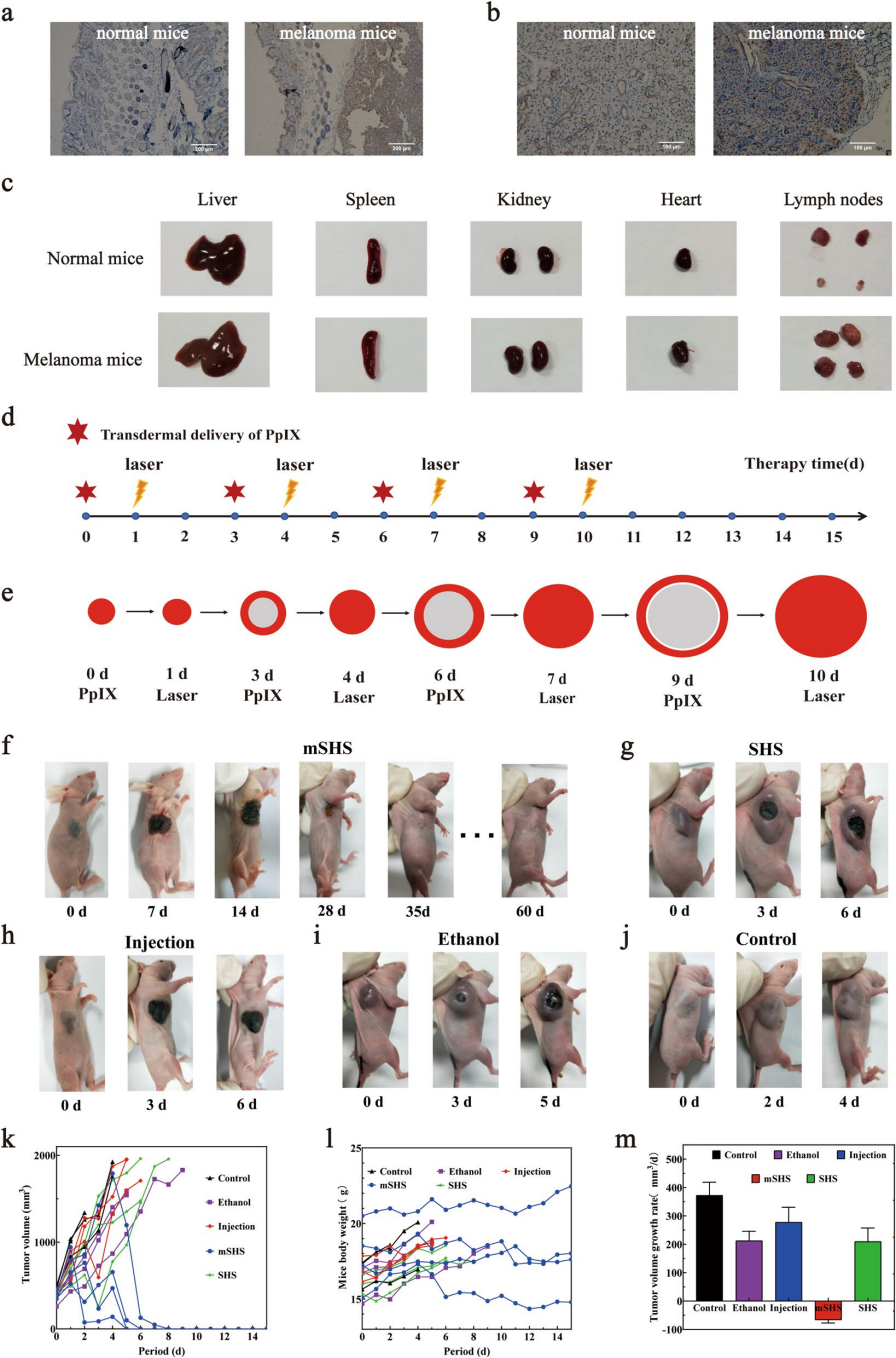
Photodynamic therapy using mSHS@PpIX completely eradicated primary melanoma in vivo. **a** Immunohistochemical staining for S-100B on longitudinal sections of subcutaneous melanoma and the neighboring skin. **b** Immunohistochemical staining for S-100B on sections of sentinel lymph nodes. **c** Representative photos of various organs of normal mice and melanoma mice. The first line shows the organs of normal mice, and the second line shows the organs of melanoma mice (before treatment). **d**, **e** General administration plan of mSHS mediated photodynamic therapy for melanoma in mice. **d** Treatment programs. The treatment period of melanoma in mice was 16 days, and the mice in each group were treated with PpIX on day 0, day 3, day 6 and day 9, respectively. Tumors underwent irradiation at a wavelength of 635 nm, with a light dosage of 1 W administered for a duration of 15 min on day 1, day 4, day 7, and day 10 consecutively. **e** The area of massage and laser. The red, orange and gray circles were simulated tumors. The red indicated the area of each administration, the orange indicated the laser irradiation site, and the gray represented the area where the drug was no longer administered. **f**–**j** Photographs showing tumor-bearing mice subsequent to diverse treatment modalities. The mice in mSHS group survived well on the 60th day. All the mice in SHS group died during the treatment. The mice in the other groups were sacrificed on the 15th day of treatment. **k** The tumor volume changes of the mice after treatment in 16 days. **l** The relative body weight changes of the mice after treatment. **m**: The weight of tumors dissected from each group of mice after treatment

We next investigated the anti-tumor efficacy of mSHS@PpIX in vivo. We established a subcutaneous melanoma model by injecting B16 cells into mice subcutaneously. We randomly selected a tumor-bearing mouse for euthanasia and excised the tumor and adjacent skin tissue to predict the progression of melanoma. The classical melanoma markers S-100B (S100 calcium-binding protein B) diagnosed that the tumor cells had aggressively invaded the dermis (Fig. 3a). In addition, immunohistochemical staining showed that melanoma cells had invaded lymph nodes (Fig. 3b). We collected organs and tissues to investigate the potential dissemination of melanoma cells to other anatomical regions. Compared with healthy mice, the lymph nodes of tumor mice were enlarged, which may have resulted from the immune response of tumor cells to lymph node stimulation (Fig. 3c). All the results indicate that the established B16 melanoma mice have progressed to stage III or beyond. Further, we treated melanoma tumors in mice (treatment protocol schema shown in Fig. 3d and e) using different treatment strategies, including: ①Injection: tail vein injection of an ethanol solution containing PpIX; ②mSHS: topical application of mSHS@PpIX; ③SHS: topical application of SHS followed by an ethanol solution containing PpIX; ④Ethanol: topical application of an ethanol solution containing PpIX. The results revealed that the topical application of mSHS@PpIX completely eradicated the melanomas of the mice on the 7th day of treatment, leaving a scar in the application area (Fig. 3f).

No solid tumor tissue was found in mice by anatomical examination after the mSHS@PpIX treatment. The treated skin then gradually recovered over 30 days (Additional file 5: Fig. S5). The mice treated with mSHS@PpIX successfully survived without any side effects, tumor metastasis, or recurrence. In contrast, mice receiving all other treatment strategies exhibited continuous tumor growth (Fig. 3g–j and Additional file 6: Fig. S6, Additional file 7: Fig. S7, Additional file 8: Fig. S8, Additional file 9: Fig. S9). The tumors' volume (V = (tumor width^2^) × (tumor length) × 0.5) in mice from the control group increased sharply, followed by the injection group, ethanol group, and SHS group (Fig. 3k). We also found that while the body weight of the mice receiving mSHS@PpIX treatment was unchanged before and after the whole treatment, mice receiving all other treatment strategies increased rapidly (Fig. 3l), indicating that the increased body weight of mice resulted from the growth of the tumors. Moreover, tumors in mice after different treatment strategies were dissected and weighed (Fig. 3m). These tumor tissues were then sectioned using cryo-microtome and stained with H&E. The tumor tissue from the SHS group was relatively loose, while those from the injection group, the ethanol group, and the control group were compact and homogeneous (Additional file 10: Fig. S10). Although mSHS@PpIX can cure primary melanoma in mice by local administration, the treatment of distant metastatic melanoma is also a major challenge in the future due to the high degree of metastasis and spread of melanin in mice [44–46]. In future research, we can try to load multiple drugs with mSHS at the same time (e.g., PDT combined immunotherapy) to cure both primary and metastatic melanoma.

### The interaction between mSHS and skin

**Fig. 4:**
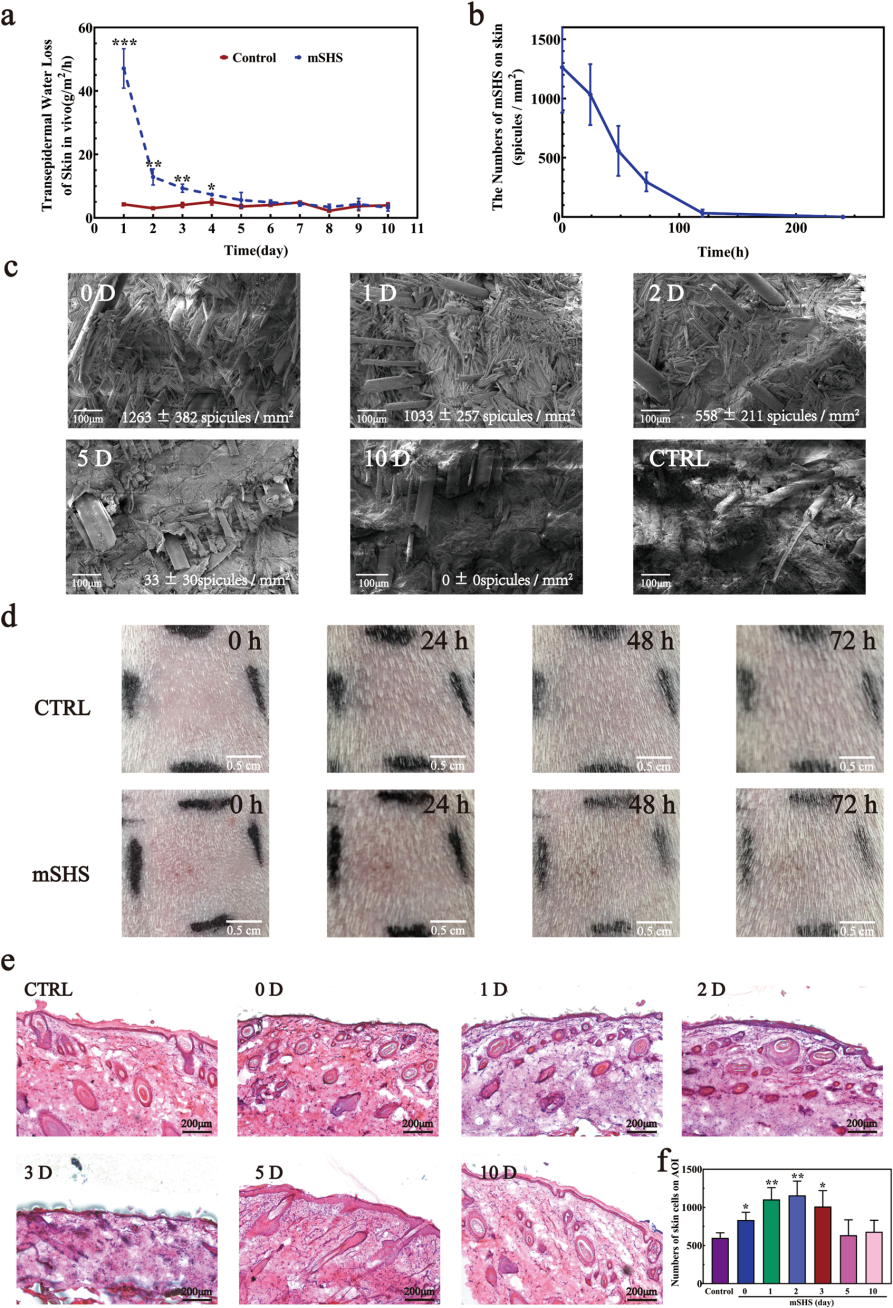
Safety evaluation of mSHS in vivo. **a**, **b** Study of the penetration behavior of mSHS on the skin of guinea pigs. **a** TEWL recovery profile of guinea pig skin following mSHS treatments was assessed over time. Values represent mean ± SD (n = 3). **b** Visualization of mSHS after its topical application. There were 1263 ± 382, 1033 ± 257, 558 ± 211, 33 ± 30 and 0 spicules per mm^2^ (n = 6) on guinea pig skin topical applied by mSHS 0, 1, 2, 5, 10 days later. And 10 days later, guinea pig skin got recovered. **c** The progression of micro-channels formation induced by mSHS treatment in vivo on guinea pig skin. Data presented as mean ± SD, n = 6. Scale bar was 100 µm. **d**, **e** Skin irritation and toxicty study of mSHS treatment in vivo. **d**: Comparison of guinea pig skin at 0 h, 24 h, 48 h, and 72 h after treatment with mSHS (10 mg per 1.77 cm^2^ administered with electrical massage for a duration of 2 min.) and the control group. Scale bar was 0.5 cm. **e** Skin cross sections with H&E stained at different time after mSHS treatment. Scale bar was 200 µm. **f** The quantity of cutaneous cells within the Area of Interest (AOI) at distinct temporal intervals subsequent to mSHS intervention. * indicates *p* < 0.05. ** indicates *p* < 0.01

Furthermore, we investigated the interactions between skin and mSHS in vivo. Firstly, we measured the transepidermal water loss (TEWL) over 240 h after the topical application of mSHS (Fig. 4a). TEWL measurement can directly reveal the skin barrier's integrity noninvasively. Without mSHS treatment, TEWL was stable (4.2 ± 0.6 g/m^2^/h) during the observation period. After applying mSHS, TEWL immediately increased to 47.1 ± 6.2 g/m^2^/h and dropped quickly to 12.9 ± 2.5 g/m^2^/h within 24 h. Then it gradually decreased to 5.6 ± 2.4 g/m^2^/h (not significant compared to control, *p* > 0.05) within the next 96 h. We also visualized mSHS insertion and distribution in guinea pig skin in vivo for 240 h (Fig. 4b&c). After the topical application, mSHS could penetrate the skin up to a depth of 62.07 ± 6.29 nm (n = 100) with a surface distribution of 1263 ± 382 (n = 6). Subsequently, mSHS gradually peeled off the skin in the next 120 h (Fig. 4c) through natural desquamation. In addition, studies have confirmed that silicon-based mesoporous materials can dissolve or degrade entirely into non-toxic silicic acid in an aqueous solution, the predominant form of silicon in the human body, and can be absorbed by the body [47], indicating that the surface mesoporous material of mSHS is safe (non-toxic) and biocompatible. We also observed the skin erythema and edema degrees induced by mSHS treatment in vivo, then evaluated its skin irritation using the primary irritation index (P.I.I), where 0–0.4 and 0.5–1.9 represent non-irritating and slightly irritating, respectively. The mSHS at a dosage of 5 mg/cm^2^ resulted in slight irritation over 24 h (P.I.I = 1.0), and the induced erythema gradually faded out in the next 48 h (Fig. 4d). We did not observe any morphological changes in keratinocytes after applying mSHS for 240 h (Fig. 4e). However, we found an obvious infiltration of immune cells (leukocytes or granulocytes) in the mSHS treatment area, which could last 120 h (Fig. 4e and f) and could further facilitate the PDT treatment.

In this study, we fabricated the mesoporous layer coated SHS (mSHS) to significantly increase the application dosage of hydrophobic drugs and also dramatically enhance their delivery into deep skin layers. Topical delivery of PpIX using mSHS (mSHS@PpIX) completely cured the primary melanoma in mice without recurrence. In the future, mSHS could be a promising topical drug delivery platform for the treatment of diverse cutaneous ailments, such as metastatic melanoma.

## Materials and methods

### Chemicals and animals

Protoporphyrin IX, coumarin 6, thiazolyl blue tetrazolium bromide (MTT), and dimethyl sulfoxide were acquired from Sigma-Aldrich. Hexadecyltrimethylammonium bromide (CTAB) and Tetraethyl Orthosilicate (TEOS) were acquired from TCI (Shanghai) Development Co., Ltd. (Shanghai, China). Chloral hydrate was obtained from Macklin (Shanghai, China). Neutral balsam and Tween 80 were procured from Solarbio (Beijing, China). Penicillin/Streptomycin Solution and Trypsin 0.25% solution were acquired from HyClone (USA). RPMI Medium 1640 basic and Fetal Bovine Serum (FBS) were purchased from Gibco (Grand Island, USA). B16 cells were procured from the Cell Bank of the Chinese Academy of Sciences (Shanghai, China). All other chemicals (analytical grade) used in this study were purchased from Sinopharm Group Co. Ltd (Shanghai, China).

Full thickness porcine skin was obtained from YinXiang Group (Xiamen, Fujian, China). Female nude mice (4–5 weeks old) were acquired from Shanghai SLAC Laboratory Animal Co., Ltd. (Shanghai, China). All in vivo experiments conducted in this research adhered to the protocols approved by the Institutional Animal Care and Use Committee at Xiamen University Laboratory Animal Center (XMULAC20210003).

### Preparation and characterization of mSHS

#### Preparation of mSHS

The sponge *Haliclona* sp. spicules (SHS) were derived from cultured sponge (*Haliclona* sp.) obtained from Dongshan Bay (Fujian, China). Subsequently, they were extracted, prepared, and purified following the methods outlined in our patent (ZL201610267764.6). Specifically, SHS (100 mg) were washed with NaOH (5 mL, 0.1 mol/L) and HCl (5 mL, 0.1 mol/L) successively in an ultrasonic cleaner for 30 min and then cleaned with deionized water. Subsequently, SHS were ultrasonically cleaned for 30 min using 5 mL acetonitrile, and finally lyophilized with a vacuum freeze dryer. To prepare mSHS, SHS (100 mg) were mixed with deionized water (35 mL), CTAB (80 mg), ammonium hydroxide (5 μL, 25 wt%) and ethanol (15 mL) under stirring for 30 min. TEOS (40 μL) was then added dropwise to the above mixture, followed by stirring at 60 ℃ for 24, 48 or 72 h. Finally, the obtained mSHS were purified by repeated centrifugation (2000 g for 10 min) and washing with deionized water to remove the by-products and then aged overnight at 100 ℃. Finally, to remove the CTAB, mSHS were placed into an ethanolic solution of ammonium nitrate and refluxed for 12 h at 80 ℃ [48–50].

#### Visualization of mSHS

The morphologies and structures of SHS and mSHS were first characterized by a scanning electron microscope (SEM, FEI Quanta 650 FEG, USA). The SHS and mSHS underwent platinum deposition using a sputter coater, resulting in the formation of a thin platinum layer. (JEOL JFC1600, Akishima, Japan). SEM micrographs were taken at an accelerating voltage of 10 kV. The SHS and mSHS were then visualized using a transmission electron microscope (TEM, FEI Tecnai G2 Spirit BioTwin, USA). Prior to analysis, the samples were deposited on copper grids. The samples were analyzed using transmission electron microscopy. Nitrogen adsorption and desorption isotherm were acquired using a Micromeritics ASAP 2010 M system. Prior to measurement, the samples were subjected to degassing at 350℃ for 24 h, and the measurements were conducted at 77 K. Pore size distributions and specific surface areas were calculated from the adsorption branches of the N2 adsorption isotherms utilizing the Barrett Joyner Halenda (BJH) and Brunauer Emmett Teller (BET) methods [31, 51].

#### The drug loading of mSHS

The method of organic solvent immersion [52] was used to load drugs into the mesoporous layer of mSHS. Briefly, Coumarin 6 or PpIX solutions (1, 2, 5, 10, 20, 30 and 40 mg/mL) were dissolved in dichloromethane. Then, 600 μL of Coumarin 6 or PpIX solution at different concentrations was mixed with 10 mg of mSHS. The mixture was shaken for 12 h using a rotating mixer (QB-210, Haimen Kylin-Bell Lab Instruments Co., Ltd., China). Afterwards, the mixture of mSHS and drug solution was centrifuged, and the excess drug solution was removed. Finally, the drug-loaded mSHS was lyophilized using a vacuum freeze dryer to obtain mSHS loaded with the drug (mSHS@drug, including mSHS@coumarin 6 or mSHS@PpIX). For in vitro or in vivo experiments, PpIX was dissolved in N,N-Dimethylformamide to prepare the PpIX solution, which was also mixed with mSHS (10 mg).

To determine the drug loading capacity of mSHS, 10 mL of ethanol was added to 10 mg of mSHS@drug, which was placed in a shaker for 12 h (28 ℃, 200 rpm). Subsequently, the concentration of coumarin 6 (excitation/emission wavelength = 466 nm/504 nm) or the concentration of PpIX (excitation/emission wavelength = 409 nm/633 nm) in the ethanol solution was determined using a microplate reader. The accuracy of all standard curves was confirmed through the verification of linearity (R^2^ = 0.9999). The drug loading capacity of mSHS was determined by employing the subsequent formula:

$${\text{Drug loading of mSHS }}\left( {\mu {\text{g }}/{\text{ mg}}} \right) \, = {\text{ C }}*{\text{ V }}/{\text{ W}};$$

C represents the concentration of durg in ethanol; V represents the volume of the ethanol (10 mL); W represents the weight of mSHS@drug (10 mg).

To visualize mSHS@drug, an appropriate amount of the powders was distributed on a glass slide in a single layer. Then, mSHS@coumarin 6 (excitation/emission wavelength = 466 nm/504 nm) or mSHS@PpIX (excitation/emission wavelength = 409 nm/633 nm) was observed using a confocal microscope (LSM780NLO, Carl Zeiss, Germany).

#### The drug release behavior of mSHS@drug

The mSHS@drug (PpIX or Coumarin 6) was selected to study the drug release behavior. In brief, a certain amount of mSHS@drug (10 mg) was dispersed into 1L of PBS (200 mM, pH = 7.4) containing 0.5% Tween80. The system was kept away from light and stirred appropriately (600 rpm). Then, 200 μL of the solution was taken at 0, 1, 2, 4, 6, 10, 12, and 24 h, respectively. Following each sampling, the system was supplemented with 0.2 mL of PBS solution containing 0.5% Tween 80. The concentration or content of the drug (PpIX or coumarin 6) in the solutions was assessed utilizing a microplate reader.

### Skin drug delivery using mSHS in vitro

#### Skin penetration study in vitro

Full thickness porcine skin was used for the skin penetration study in vitro. Briefly, the subcutaneous adipose tissue beneath the dermis was meticulously excised using a scalpel, while the porcine hair shaft was cut off with the length shorter than 2 mm employing an electric razor. Subsequently, the treated skin was rinsed with deionized water and subsequently stored at a temperature of − 20 °C. Prior to usage, the porcine skin was retrieved from the refrigerator and allowed to thaw at a temperature of 25 °C. The in vitro skin penetration test was performed using Franz diffusion cells (ZhengTong Co. Ltd., China). The skin was punched out in disk samples (3.6 cm). The skin was placed on top of the vertical Franz diffusion cells with the epidermis facing the donor compartment. The receptor compartment was filled with PBS (12 mL, 0.2 M) containing 1% Tween80. The effective penetration area of the diffusion cells was 1.77 cm^2^. All bubbles between the receptor solution and the skin are completely removed, and then put it in the water bath of 36.5 ~ 37.5 ℃ to maintain the skin surface temperature at 36.5 ~ 37.5 ℃. The skin's conductivity was assessed to evaluate the integrity of the skin barrier according to the method described in previous studies [36].

The application of SHS or mSHS (100 μL, 100 mg/mL) was achieved by massaging (electric massage). The force applied to the skin surface during the massage was about 0.3 N with the application time of 120 s (300 r/min). After the massage, the residual SHS was removed by washing the skin surface with PBS (200 mM) for 3 times. The drug solution (saturated solution of coumarin 6 or PpIX in 30% ethanol, 200 μL) was then topically applied on the surface of the skin. Each experimental formulation underwent triplicate evaluations. All the groups were performed occlusively for 16 h.

At the conclusion of the skin penetration experiment, 1 mL of the solution was extracted from the receptor compartment. Subsequently, the skin surface underwent three washes using PBS.

The experimental method of tape-stripping to remove SC has been described in our previous studies [36, 37]. The SC layers were subjected to ten successive stripping procedures for their removal: The first strip isolated SC 1, while SC 2 was obtained from the second to fifth strips. SC 3 was obtained from the sixth to tenth strips. The viable epidermis layer was surgically separated from the dermis using a scalpel. Subsequently, the remaining dermis layer was fragmented into diminutive fragments. Methanol was employed for the extraction of coumarin 6 (or PpIX) from individual skin layers. The extraction solution was taken out and centrifuged (8000 r/min, 5 min) to precipitate the skin tissue, and the resulting supernatants were collected. Subsequently, the concentration of coumarin 6 (excitation/emission = 466/504 nm) or PpIX (excitation/emission = 409/633 nm) in the receptor phase and various skin layers was assessed by using microplate reader.

#### Visualization of skin drug penetration

The skin penetration of drug (coumarin 6 or PpIX) was also visualized using confocal microscopy. After the skin penetration experiment in vitro, a small piece of skin measuring 2.5 mm in radius was extracted by punching and promptly preserved in the OCT compound (Sakura Finetek, USA) rapidly. The porcine skin was then cut into a section of 10 μm. Subsequently, the skin section was placed onto an adhesive glass slide, neutral balsam (50 μL) was added to the skin, and a cover slip was then positioned on top. The resulting sample was visualized using a confocal microscope (Carl Zeiss, LSM780NLO, Jena, Germany). The parameters of the confocal microscopy system remained constant for all samples.

#### Cytotoxicity of PpIX

B16 cells (mouse melanoma cell) were cultivated in RPMI Medium 1640 basic (1 ×) supplemented with 1% 100 × Penicillin/Streptomycin Solution and 10% FBS (fetal bovine serum, Gibco). The cells were incubated at 37 °C in a humidified incubator with 5% CO2. The B16 cells were plated in 96-well plates at a density of 1 × 10^4^ cells/well and cultured for a duration of 24 h. PpIX was dissolved in DMSO at 1 mg/mL and then diluted with water to different concentrations using cell culture media (containing 1% antibiotics and10% FBS). The cells were then incubated with PpIX at different concentration for 6 h away from light. Afterwards, the cell culture medium in 96 well plate was replaced with PBS (0.01 M, pH = 7.4) and B16 cells were exposed to 635 nm radiation at a light dosage of 5 J/cm^2^ for 10 min. After the irradiation, the PBS solution was replaced with cell culture medium and the cells were incubated in an incubator (37 ℃, 5% CO_2_) for 24 h in the dark. Thereafter, the cell viability was evaluated utilizing the MTT assay. Finally, the cytotoxicity of different concentrations of PpIX to cells were calculated according to the absorbance.

The internalization of PpIX in cells over time was visualized using a confocal microscope at different time points (2, 4, 6 and 8 h). In brief, B16 cells were inoculated into 96-well plates at a density of 1 × 10^4^ cells/well and subjected to a 24 h incubation period. Following that, the cells were exposed to PpIX at a concentration of 1 μg/mL. Afterward, the culture medium was aspirated, and the cells underwent three washes with PBS. Subsequently, 0.1 mL of PBS was added to each well, and the distribution of fluorescence was examined using a confocal microscope.

### Evaluation of mSHS treatment in vivo

#### *Evaluation of mSHS efficacy in a melanoma mouse model *in vivo

Female nude mice were used to develop the metastatic melanoma mouse model. Briefly, the right forelimb of nude mice underwent sterilization using alcohol, followed by subcutaneous injection of B16 cells into the aforementioned limb of nude mice(4–5 weeks old). The tumor's maximum dimension (L) and minimum dimension (W) were assessed daily using a slide caliper to track tumor progression. Tumor volume can be determined using the formula: V = (tumor width^2^) × (tumor length) × 0.5. When the tumor volume reached about 500 mm^3^, a randomly chosen mouse bearing the tumor was selected to evaluate the progression of melanoma.

The mice harboring tumors were subsequently allocated randomly into five distinct experimental groups (4 animals per group), including (1) mSHS group: The tumor of mice was subjected to an even application of mSHS@PpIX (5.6 mg/cm^2^) for a duration of 3 min, utilizing an applied force of 0.3 N; (2) SHS group: The tumor of mice was subjected to an even application of SHS (5.6 mg/cm^2^) for a duration of 3 min, utilizing an applied force of 0.3 N. Then a saturated PpIX solution (ethanol) of 200 μL was topically applied to the tumor surface; (3) Injection group: the saturated PpIX ethanol solution (200 μL) was slowly injected into mice by tail intravenous injection; (4) Ethanol group: the saturated PpIX ethanol solution (200 μL) was topically applied to the tumor surface; (5) Control group: the tumor was not treated with PpIX administration and only treated with irradiation.

The treatment period of melanoma in mice was 16 days, and the first day of treatment was recorded as day 0. Before treatment, one tumor-bearing mice was randomly selected from each group to be sacrificed and the tumor was collected. The rest of the mice were treated four times, that is, on day 0, day 3, day 6 and day 9, respectively (Additional file 10: Fig. S10).

The tumors were exposed to 635 nm irradiation at a light intensity of 1 W for a duration of 15 min on day 1, day 4, day 7, and day 10, respectively. The spot size is adjusted to match the size of the tumor, and non-tumor areas are protected from light with aluminum foil. The body weight and tumor volume of the mice were assessed on a daily basis throughout the course of the treatment. After treatment for 16 days, the mice were sacrificed and tumor tissues were peeled off. The tumors were collected before and after treatment, and the tumor tissues underwent sectioning and were subjected to H&E staining. Mice received intraperitoneal injections of pentobarbital sodium for anesthesia during treatment.

#### Predicting the progression of melanoma

A tumor-bearing mouse was randomly selected for euthanasia, the tumor and its adjacent skin tissue were excised to predict the progression of melanoma. Subsequently, organs and tissues, including the liver, heart, spleen, lymph nodes, and kidneys, were collected from mice harboring tumors and normal mice to examine the potential dissemination of melanoma cells to other parts of the body. The tumor and skin specimens were immobilized in a 4% paraformaldehyde solution. Following a 48-h fixation period, all tissue samples were encased in paraffin blocks. Subsequently, sections measuring 5 µm in thickness were obtained from each block using a paraffin cutter (RM2128, Leica, Germany), and stained with immunohistochemical markers (S-100B) for microscope observation (Carl Zeiss, Axio Imager A2, Jena, Germany).

### Safety assessment of mSHS in vivo

#### Measurement of transepidermal water loss (TEWL)

TEWL was measred to study the effect of mSHS on the skin barrier and the recovery time of skin microchannels in guinea pigs in vivo. Briefly, all female guinea pigs (12 weeks old) were subjected to ether inhalation for anesthesia and the back hair of the guinea pigs was clipped. Further, according to the experimental needs, appropriate regions were selected on the back for numbering and grouping, and three parallel data were set for each group. In the mSHS group, mSHS (10 mg/1.77 cm^2^) was applied with a massage for 2 min by an electric massager. TEWL measurements were performed over 10 days after mSHS treatment and continued for a period of time until measurements were returned to baseline level.

#### Skin irritation test

The skin irritation induced by mSHS treatment was assessed using guinea pigs. Following a one-week acclimation period, the guinea pigs underwent anesthesia using ether inhalation and the back hair of the guinea pigs was clipped. Further, according to the experimental needs, appropriate regions were selected on the back for numbering and grouping. mSHS (10 mg/1.77 cm^2^) was applied on back skin of guinea pigs using an electric massager, applying gentle massage for a duration of 2 min. Subsequently, the degree of erythema and edema of each area was observed and recorded at 0 h, 24 h, 48 h, and 72 h after mSHS treatment. The calculation of the Primary Irritation Index (P.I.I.) was based on the following equation:

$${\text{P}}.{\text{I}}.{\text{I}}.=\frac{\sum \mathrm{Erythema\, grade\, at\, }1{\text{h}}, 24\mathrm{h\ and\, }72{\text{h}}+\sum \mathrm{Edema\, grade\ at\, }1{\text{h}}, 24\mathrm{h\ and\, }72{\text{h}}}{\mathrm{number\, of\, guinea\, pigs}\times \mathrm{time\, of\, reading}\times \mathrm{number\, of\, application\ skin\, sites}}$$

The potential for skin irritation caused by the mSHS was assessed using the Draize dermal scoring criteria, which are explained as follows. The criteria employed for Draize dermal scoring.

**Table:** 

P.I.I	Classification
0.0–0.4	No irritation
0.5–1.9	Slight irritation
2.0–4.9	Moderate irritation
5.0–8.0	Severe irritation

In addition, skin specimens from both treated and untreated regions were acquired at 0, 1, 2, 3, 5, and 10 days after the mSHS application. Before collecting skin samples, the guinea pigs were killed. The skin samples obtained were cryopreserved in OCT medium and sliced into sections of 10 μm thickness using a freezing microtome (CM1900, Leica). The samples were immobilized using a 10% formaldehyde solution, stained with hematoxylin and eosin, and then photographed with an optical microscope. The quantification of skin cells within the Area of Interest (AOI) were conducted using Image-Pro Plus 6.0 software.

#### Scanning electron microscopy (SEM) study

Skin samples were obtained from both treated and untreated areas at different time points following the application of mSHS. The collected skin samples were immersed in a 2.5% glutaraldehyde solution for a duration of 2 h. Subsequently, they were subjected to three consecutive washes, each lasting for 10 min, using a 100 μM PBS solution with a pH of 7.4. The samples underwent dehydration at 4 ℃ using various ethanol concentrations for a duration of 15 min and then transferred to 25 ℃, tertiary butanol was used to replace ethanol. After soaking the sample in tert-butanol for 3 times (10 min each time), the sample was further freeze-dried. Finally, skin samples underwent a process of platinum (30 nm) coating utilizing a Sputter Coater, followed by observation through a SEM (FEI Quanta 650 FEG, USA).

### Statistical analysis

Statistical significance was assessed using the two-tailed and unpaired Student’s t-test in Microsoft Excel. The data in this study was reported as mean ± standard deviation (SD). A minimum of three independent samples were examined in all experimental analyses. The *p* values < 0.05 are considered to be significantly different.

### Supplementary Information

**Additional file 1: Fig S1**. (a): Photo of Sponge *Haliclona sp*. (b): Photo of SHS.

**Additional file 2: Fig S2.** (a): SEM images of mSHS after 24 h modification. (b): SEM images of mSHS after 48 h modification. (c): SEM images of mSHS after 72 h modification. (d): Mesoporous layer thickness at different times. (e): Visualization of mSHS@ PpIX and mSHS@ FITC-dextran (MW=10K) (Scale bar was 100 µm). Equal amounts of mSHS@PpIX and mSHS@ FITC-dextran were mixed, and the mixture was visualized by confocal microscopy.

**Additional file 3: Fig S3. **PpIX loading and release behavior of mSHS. (a)Drug loading of mSHS at different concentration of the PpIX. (c): Release behavior of mSHS@PpIX.

**Additional file 4: Fig S4.** Coumarin 6 loading and release behavior of mSHS. (a): Drug loading of mSHS at different quality ratios of coumarin 6 and mSHS. (b): Release behavior of mSHS@ coumarin 6. The red arrow indicates that the system was shaken in the shaker for 24h (25℃, 180 rpm) after standing for 24 h.

**Additional file 5: Fig S5. **Photographs of tumor-bearing mice in the mSHS group during treatment.

**Additional file 6: Fig S6. **Photographs of tumor-bearing mice in the SHS group during treatment. The mice in SHS group died on the 9th day during the treatment.

**Additional file 7: Fig S7.** Photographs of tumor-bearing mice in the Injection group during treatment.

**Additional file 8: Fig S8.** Photographs of tumor-bearing mice in the Ethanol group during treatment.

**Additional file 9: Fig S9.** Photographs of tumor-bearing mice in the control group (untreated).

**Additional file 10: Fig S10.** H&E analysis of B16 tumors dissected from mice before (0 d) and after (15 d) treatment. Scale bar was 50 µm.

## Data Availability

The data presented in this study are available on request from the corresponding author.

## References

[1] Liu JY, Lowe M (2019). Neoadjuvant treatments for advanced resectable melanoma. J Surg Oncol.

[2] Baldea I, Filip AG (2012). Photodynamic therapy in melanoma—an update. J Physiol Pharmacol.

[3] Teramoto Y, Keim U, Gesierich A, Schuler G, Fiedler E, Tuting T, Ulrich C, Wollina U, Hassel JC, Gutzmer R, Goerdt S, Zouboulis C, Leiter U, Eigentler TK, Garbe C (2018). Acral lentiginous melanoma: a skin cancer with unfavourable prognostic features. A study of the German central malignant melanoma registry (CMMR) in 2050 patients. Br J Dermatol.

[4] Rausei S, Pappalardo V, Boni L, Dionigi G (2018). Laparoscopic intragastric resection of melanoma cardial lesion. Surg Oncol.

[5] Chaput L, Laurent E, Pare A, Sallot A, Mourtada Y, Ossant F, Vaillant L, Patat F, Machet L (2018). One-step surgical removal of cutaneous melanoma with surgical margins based on preoperative ultrasound measurement of the thickness of the melanoma. Eur J Dermatol.

[6] Wollina U, Brzezinski P (2019). The value of metastasectomy in stage IV cutaneous melanoma. Wien Med Wochenschr.

[7] Luke JJ, Schwartz GK (2013). Chemotherapy in the management of advanced cutaneous malignant melanoma. Clin Dermatol.

[8] Yang AS, Chapman PB (2009). The history and future of chemotherapy for melanoma. Hematol Oncol Clin North Am.

[9] Kawabe M, Mori T, Ito Y, Murakami M, Sakai H, Yanai T, Maruo K (2015). Outcomes of dogs undergoing radiotherapy for treatment of oral malignant melanoma: 111 cases (2006–2012). J Am Vet Med Assoc.

[10] Uslu U, Schliep S, Erdmann M (2018). Rapid development of cutaneous melanoma metastases after herpes zoster infection in a radiotherapy field. Clin Exp Dermatol.

[11] Shi W (2015). Role for radiation therapy in melanoma. Surg Oncol Clin N Am.

[12] Rodriguez-Cerdeira C, Carnero Gregorio M, Lopez-Barcenas A, Sanchez-Blanco E, Sanchez-Blanco B, Fabbrocini G, Bardhi B, Sinani A, Guzman RA (2017). Advances in immunotherapy for melanoma: a comprehensive review. Mediat Inflamm.

[13] Orloff M (2018). Melanoma immunotherapy in the elderly. Curr Oncol Rep.

[14] George DD, Armenio VA, Katz SC (2017). Combinatorial immunotherapy for melanoma. Cancer Gene Ther.

[15] Mandala M, Rutkowski P (2019). Rational combination of cancer immunotherapy in melanoma. Virchows Arch.

[16] Li Z, Wang C, Deng H, Wu J, Huang H, Sun R, Zhang H, Xiong X, Feng M (2019). Robust photodynamic therapy using 5-ALA-incorporated nanocomplexes cures metastatic melanoma through priming of CD4(+)CD8(+) double positive T cells. Adv Sci.

[17] Castano AP, Mroz P, Hamblin MR (2006). Photodynamic therapy and anti-tumour immunity. Nat Rev Cancer.

[18] Agostinis P, Berg K, Cengel KA, Foster TH, Girotti AW, Gollnick SO, Hahn SM, Hamblin MR, Juzeniene A, Kessel D, Korbelik M, Moan J, Mroz P, Nowis D, Piette J, Wilson BC, Golab J (2011). Photodynamic therapy of cancer: an update. CA Cancer J Clin.

[19] Naidoo C, Kruger CA, Abrahamse H (2018). Photodynamic therapy for metastatic melanoma treatment: a review. Technol Cancer Res Treat.

[20] Kawczyk-Krupka A, Bugaj AM, Latos W, Zaremba K, Sieron A (2013). Photodynamic therapy in treatment of cutaneous and choroidal melanoma. Photodiagnosis Photodyn Ther.

[21] Naidoo C, Kruger CA, Abrahamse H (2019). Simultaneous photodiagnosis and photodynamic treatment of metastatic melanoma. Molecules.

[22] Prausnitz MR, Mitragotri S, Langer R (2004). Current status and future potential of transdermal drug delivery. Nat Rev Drug Discov.

[23] Brown MB, Martin GP, Jones SA, Akomeah FK (2006). Dermal and transdermal drug delivery systems: current and future prospects. Drug Deliv.

[24] Engelke L, Winter G, Hook S, Engert J (2015). Recent insights into cutaneous immunization: How to vaccinate via the skin. Vaccine.

[25] Elias MD, Peter M (1983). Epidermal lipids, barrier function, and desquamation. J Investig Dermatol.

[26] Cevc G, Vierl U (2010). Nanotechnology and the transdermal route: a state of the art review and critical appraisal. J Controlled Release.

[27] Kwiatkowski S, Knap B, Przystupski D, Saczko J, Kedzierska E, Knap-Czop K, Kotlinska J, Michel O, Kotowski K, Kulbacka J (2018). Photodynamic therapy—mechanisms, photosensitizers and combinations. Biomed Pharmacother.

[28] Bacellar IOL, Oliveira MC, Dantas LS, Costa EB, Junqueira HC, Martins WK, Durantini AM, Cosa G, Di Mascio P, Wainwright M, Miotto R, Cordeiro RM, Miyamoto S, Baptista MS (2018). Photosensitized membrane permeabilization requires contact-dependent reactions between photosensitizer and lipids. J Am Chem Soc.

[29] Feng L, Cheng L, Dong Z, Tao D, Barnhart TE, Cai W, Chen M, Liu Z (2017). Theranostic liposomes with hypoxia-activated prodrug to effectively destruct hypoxic tumors post-photodynamic therapy. ACS Nano.

[30] Mitragotri S (2013). Devices for overcoming biological barriers: the use of physical forces to disrupt the barriers. Adv Drug Deliv Rev.

[31] Qu F, Zhu G, Huang S, Li S, Sun J, Zhang D, Qiu S (2006). Controlled release of captopril by regulating the pore size and morphology of ordered mesoporous silica. Microporous Mesoporous Mater.

[32] Cauda V, Mühlstein L, Onida B, Bein T (2009). Tuning drug uptake and release rates through different morphologies and pore diameters of confined mesoporous silica. Microporous Mesoporous Mater.

[33] Kapoor S, Hegde R, Bhattacharyya AJ (2009). Influence of surface chemistry of mesoporous alumina with wide pore distribution on controlled drug release. J Controlled Release.

[34] Van Speybroeck M, Mols R, Mellaerts R, Thi TD, Martens JA, Van Humbeeck J, Annaert P, Van den Mooter G, Augustijns P (2010). Combined use of ordered mesoporous silica and precipitation inhibitors for improved oral absorption of the poorly soluble weak base itraconazole. Eur J Pharm Biopharm.

[35] Gounani Z, Asadollahi MA, Pedersen JN, Lyngso J, Skov Pedersen J, Arpanaei A, Meyer RL (2019). Mesoporous silica nanoparticles carrying multiple antibiotics provide enhanced synergistic effect and improved biocompatibility. Colloids Surf B Biointerfaces.

[36] Zhang S, Ou H, Liu C, Zhang Y, Mitragotri S, Wang D, Chen M (2017). Skin delivery of hydrophilic biomacromolecules using marine sponge spicules. Mol Pharm.

[37] Zhang C, Zhang K, Zhang J-L, Ou H, Duan J, Zhang S, Wang D, Mitragotri S, Chen M (2019). Skin delivery of hyaluronic acid by the combined use of sponge spicules and flexible liposomes. Biomater Sci.

[38] Liang X, Zhang J, Ou H, Chen J, Mitragotri S, Chen M (2020). Skin delivery of sirna using sponge spicules in combination with cationic flexible liposomes. Mol Ther Nucleic Acids.

[39] Tu J, Du G, Reza Nejadnik M, Monkare J, van der Maaden K, Bomans PHH, Sommerdijk N, Slutter B, Jiskoot W, Bouwstra JA, Kros A (2017). Mesoporous silica nanoparticle-coated microneedle arrays for intradermal antigen delivery. Pharm Res.

[40] Xu B, Jiang G, Yu W, Liu D, Zhang Y, Zhou J, Sun S, Liu Y (2017). H_2_O_2_-responsive mesoporous silica nanoparticles integrated with microneedle patches for the glucose-monitored transdermal delivery of insulin. J Mater Chem B.

[41] Todo H, Kimura E, Yasuno H, Tokudome Y, Hashimoto F, Ikarashi Y, Sugibayashi K (2010). Permeation pathway of macromolecules and nanospheres through skin. Biol Pharm Bull.

[42] Sapino S, Oliaro-Bosso S, Zonari D, Zattoni A, Ugazio E (2017). Mesoporous silica nanoparticles as a promising skin delivery system for methotrexate. Int J Pharm.

[43] Stylli SS, Howes M, MacGregor L, Rajendra P, Kaye AH (2004). Photodynamic therapy of brain tumours: evaluation of porphyrin uptake versus clinical outcome. J Clin Neurosci.

[44] Rastrelli M, Tropea S, Rossi CR, Alaiba M (2014). Melanoma: epidemiology, risk factors, pathogenesis, diagnosis and classification. In Vivo.

[45] Pavri SN, Clune J, Ariyan S, Narayan D (2016). Malignant melanoma: beyond the basics. Plast Reconstr Surg.

[46] Fernandes GNC (2019). Immunomodulatory drugs in melanoma brain metastases. Discoveries.

[47] Low SP, Voelcker NH, Canham LT, Williams KA (2009). The biocompatibility of porous silicon in tissues of the eye. Biomaterials.

[48] Beck JS, Vartuli JC, Roth WJ, Leonowicz ME, Kresge CT, Schmitt KD, Chu CT-W, Olson DH, Sheppard EW, McCullen SB, Higgins JB, Schlenker JL (1992). A new family of mesoporous molecular sieves prepared with liquid crystal templates. J Am Chem Soc.

[49] Kresge CT, Leonowicz ME, Roth WJ, Vartuli C, Beck JS (1992). Ordered mesoporous molecular sieves synthesized by a liquid-crystal template mechanism. Nature.

[50] Teng Z, Zheng G, Dou Y, Li W, Mou CY, Zhang X, Asiri AM, Zhao D (2012). Highly ordered mesoporous silica films with perpendicular mesochannels by a simple stober-solution growth approach. Angew Chem Int Ed Engl.

[51] Qu F, Zhu G, Lin H, Zhang W, Sun J, Li S, Qiu S (2006). A controlled release of ibuprofen by systematically tailoring the morphology of mesoporous silica materials. J Solid State Chem.

[52] Mellaerts R, Jammaer JAG, Van Speybroeck M, Chen H, Humbeeck JV, Augustijns P, Mooter GV, Martens JA (2008). Physical state of poorly water soluble therapeutic molecules loaded into SBA-15 ordered mesoporous silica carriers: a case study with itraconazole and ibuprofen. Langmuir.

