# Supplementary figures and images for: Mesoporous silica coated spicules for photodynamic therapy of metastatic melanoma
Source: J Nanobiotechnology. 2024 Apr 15;22:179. doi: 10.1186/s12951-024-02471-y (PMC11017598; doi:10.1186/s12951-024-02471-y)

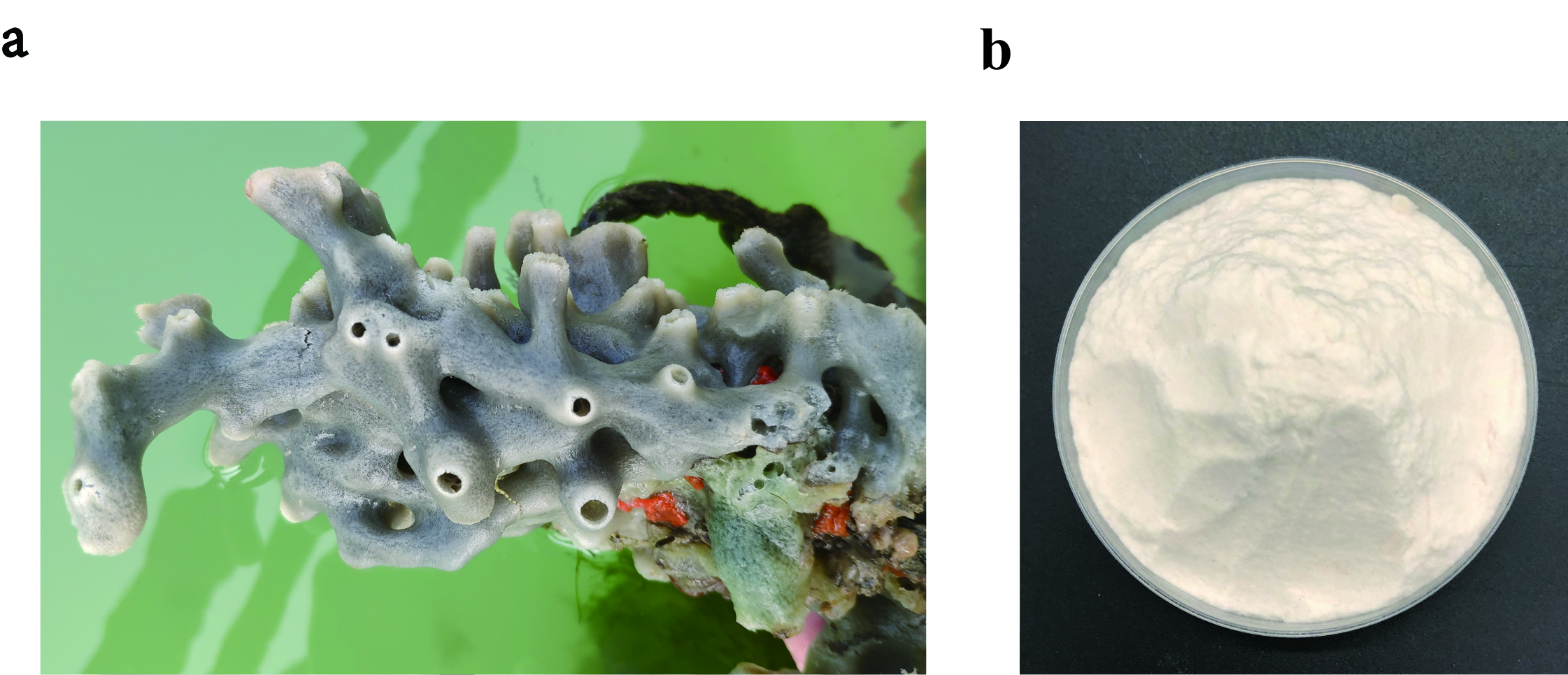

Supplement: Supplementary file 1 — Additional file 1: Fig S1. (a): Photo of Sponge Haliclona sp. (b): Photo of SHS. [file 12951_2024_2471_MOESM1_ESM.tif]

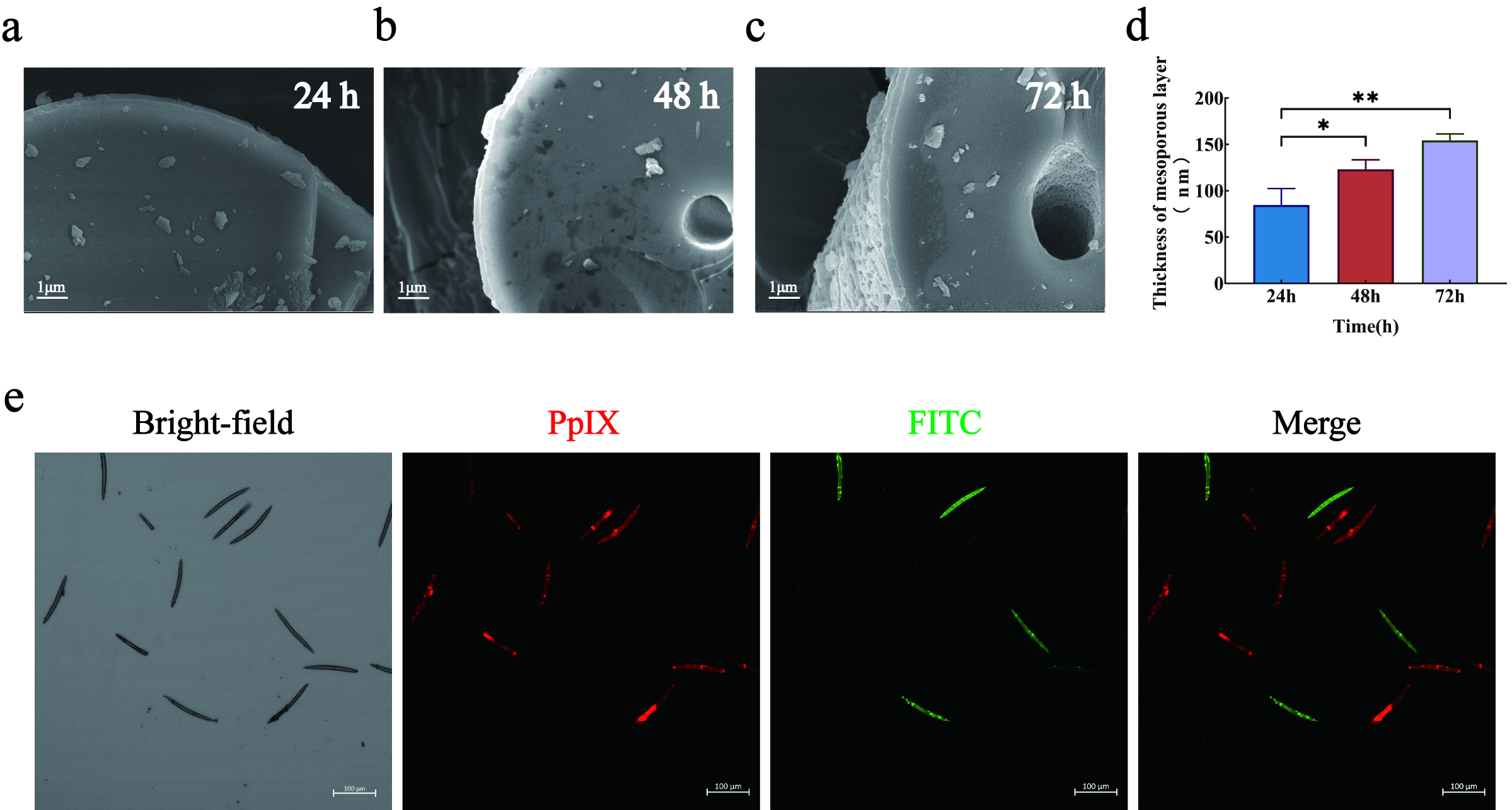

Supplement: Supplementary file 2 — Additional file 2: Fig S2. (a): SEM images of mSHS after 24 h modification. (b): SEM images of mSHS after 48 h modification. (c): SEM images of mSHS after 72 h modification. (d): Mesoporous layer thickness at different times. (e): Visualization of mSHS@ PpIX and mSHS@ FITC-dextran (MW=10K) (Scale bar was 100 µm). Equal amounts of mSHS@PpIX and mSHS@ FITC-dextran were mixed, and the mixture was visualized by confocal microscopy. [file 12951_2024_2471_MOESM2_ESM.tif]

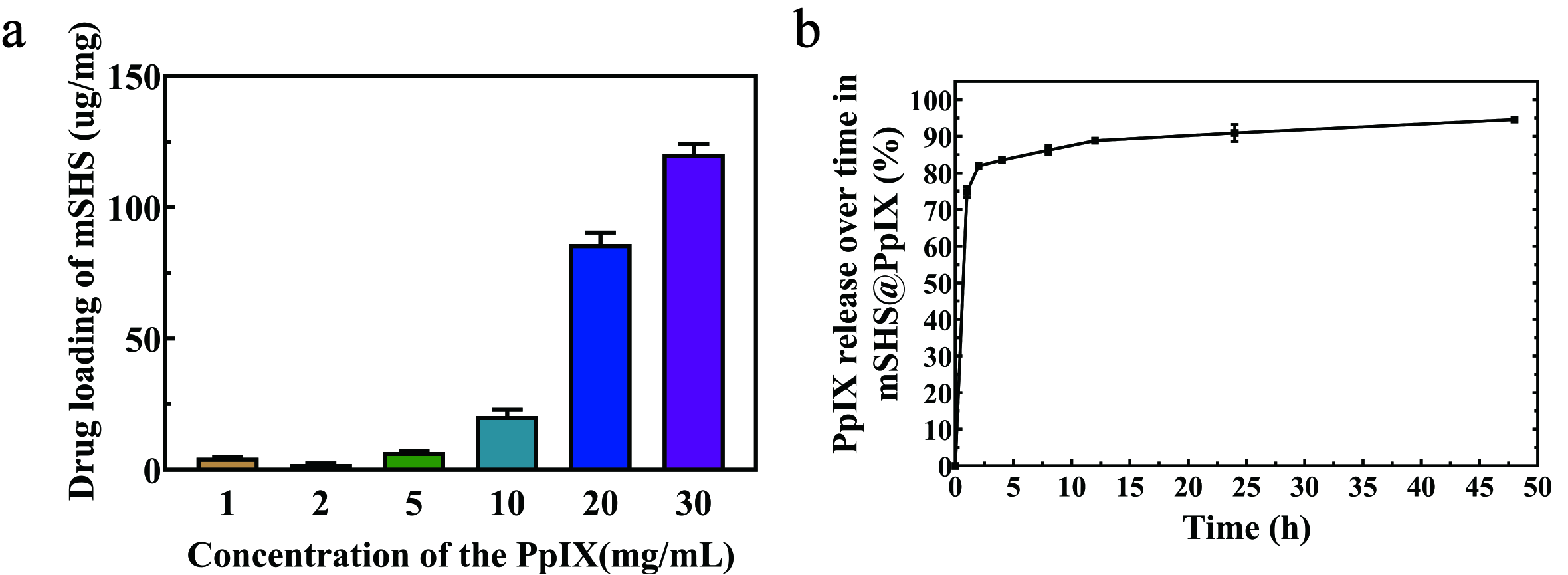

Supplement: Supplementary file 3 — Additional file 3: Fig S3. PpIX loading and release behavior of mSHS. (a)Drug loading of mSHS at different concentration of the PpIX. (c): Release behavior of mSHS@PpIX. [file 12951_2024_2471_MOESM3_ESM.tif]

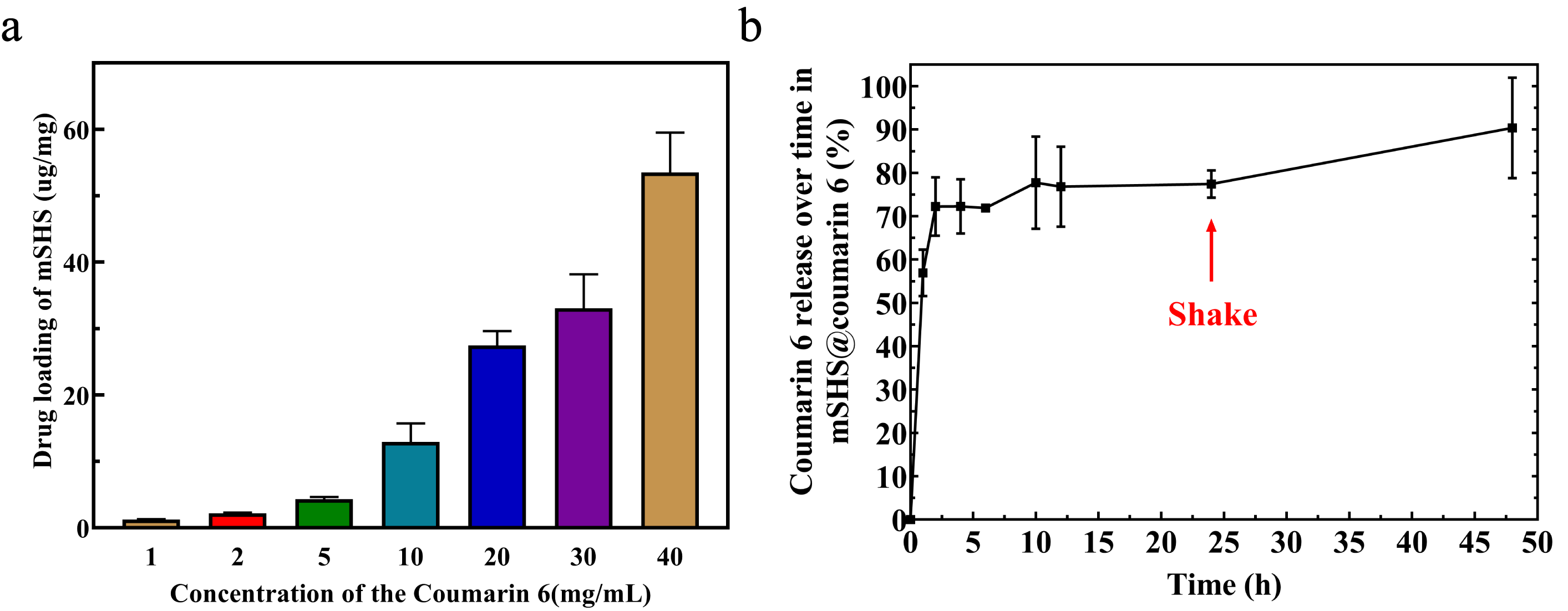

Supplement: Supplementary file 4 — Additional file 4: Fig S4. Coumarin 6 loading and release behavior of mSHS. (a): Drug loading of mSHS at different quality ratios of coumarin 6 and mSHS. (b): Release behavior of mSHS@ coumarin 6. The red arrow indicates that the system was shaken in the shaker for 24h (25℃, 180 rpm) after standing for 24 h. [file 12951_2024_2471_MOESM4_ESM.tif]

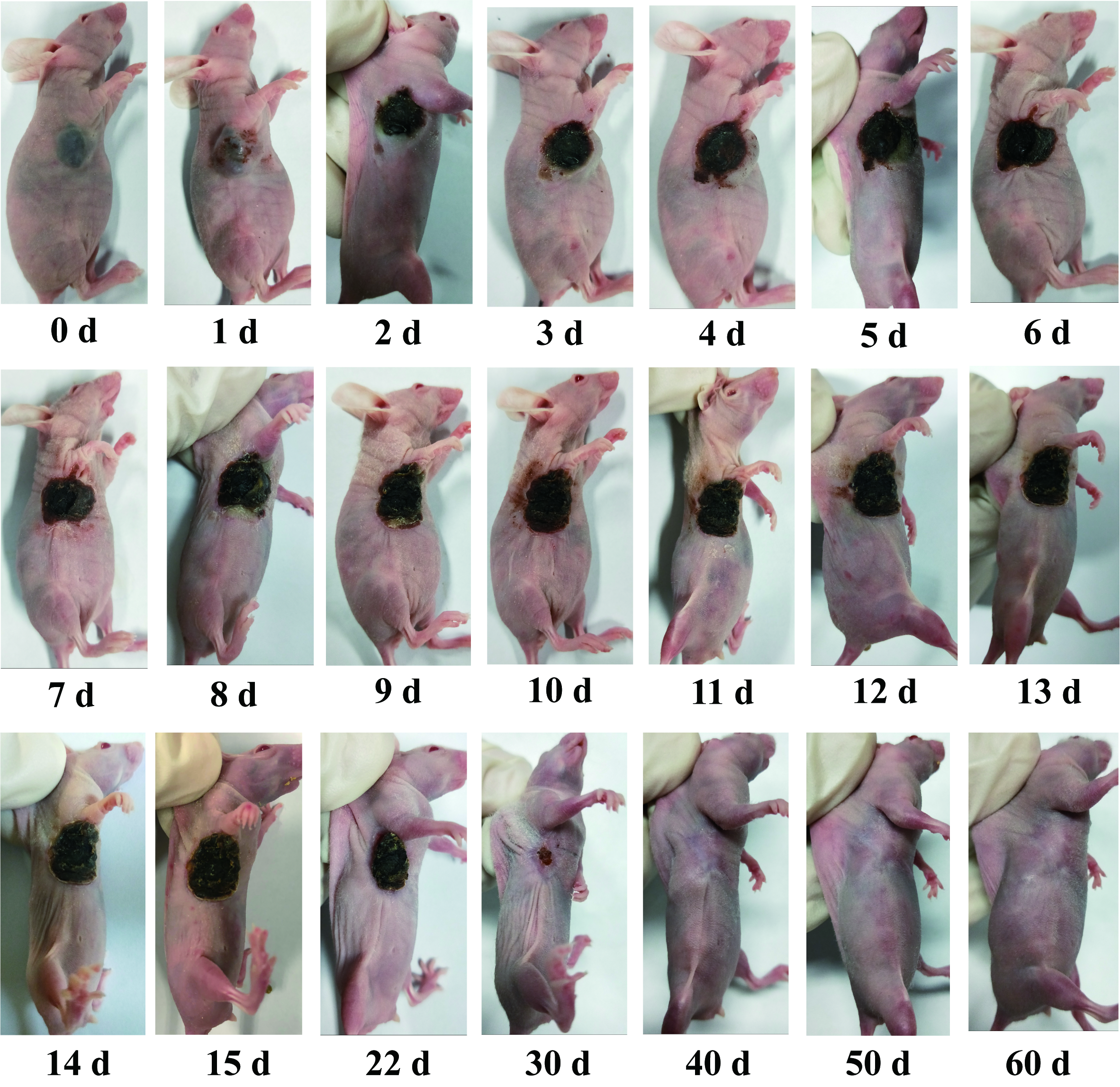

Supplement: Supplementary file 5 — Additional file 5: Fig S5. Photographs of tumor-bearing mice in the mSHS group during treatment. [file 12951_2024_2471_MOESM5_ESM.tif]

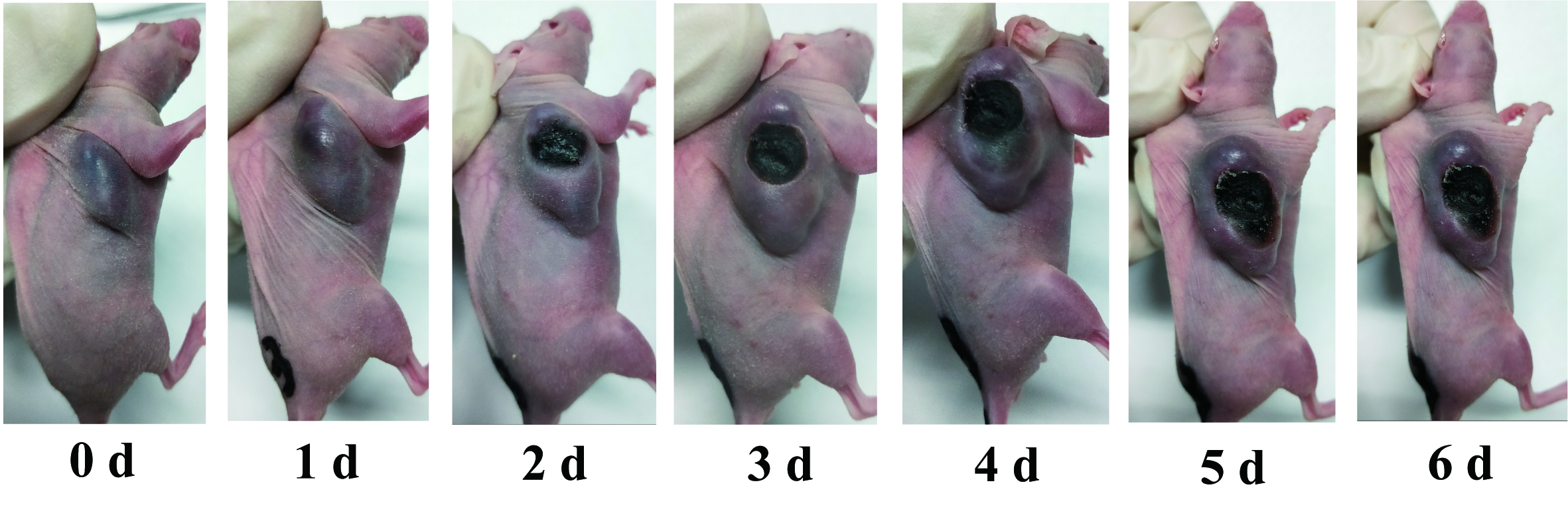

Supplement: Supplementary file 6 — Additional file 6: Fig S6. Photographs of tumor-bearing mice in the SHS group during treatment. The mice in SHS group died on the 9th day during the treatment. [file 12951_2024_2471_MOESM6_ESM.tif]

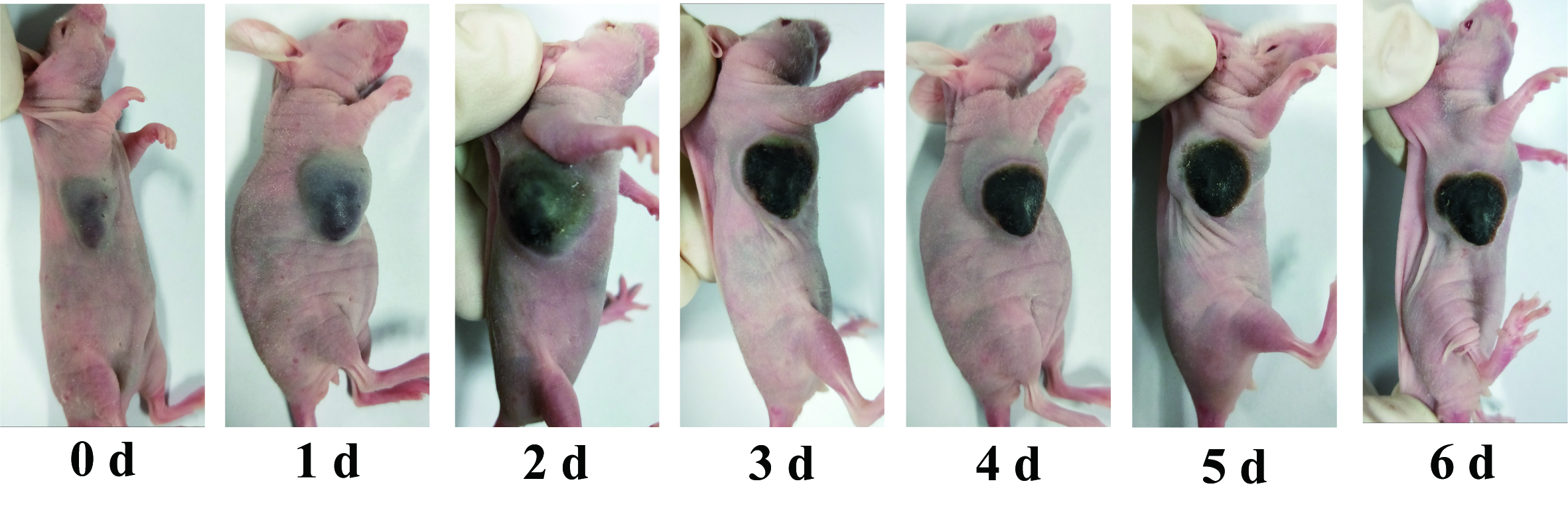

Supplement: Supplementary file 7 — Additional file 7: Fig S7. Photographs of tumor-bearing mice in the Injection group during treatment. [file 12951_2024_2471_MOESM7_ESM.tif]

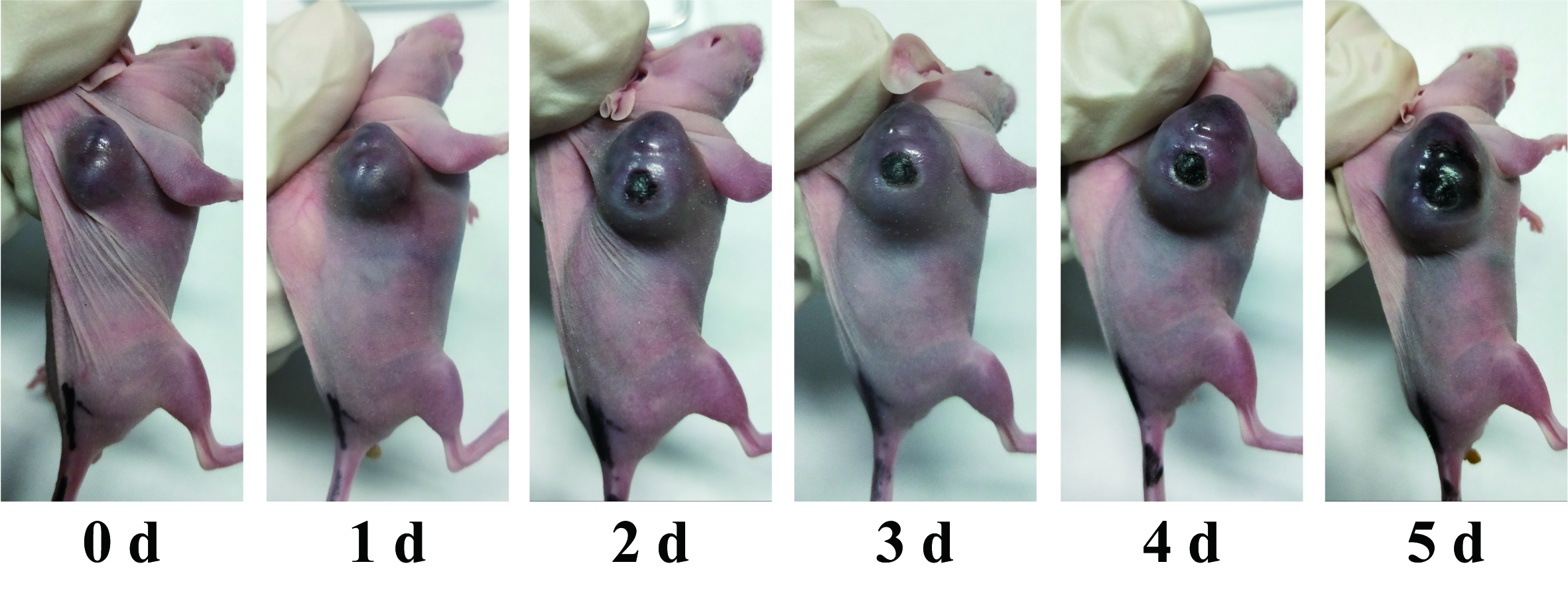

Supplement: Supplementary file 8 — Additional file 8: Fig S8. Photographs of tumor-bearing mice in the Ethanol group during treatment. [file 12951_2024_2471_MOESM8_ESM.tif]

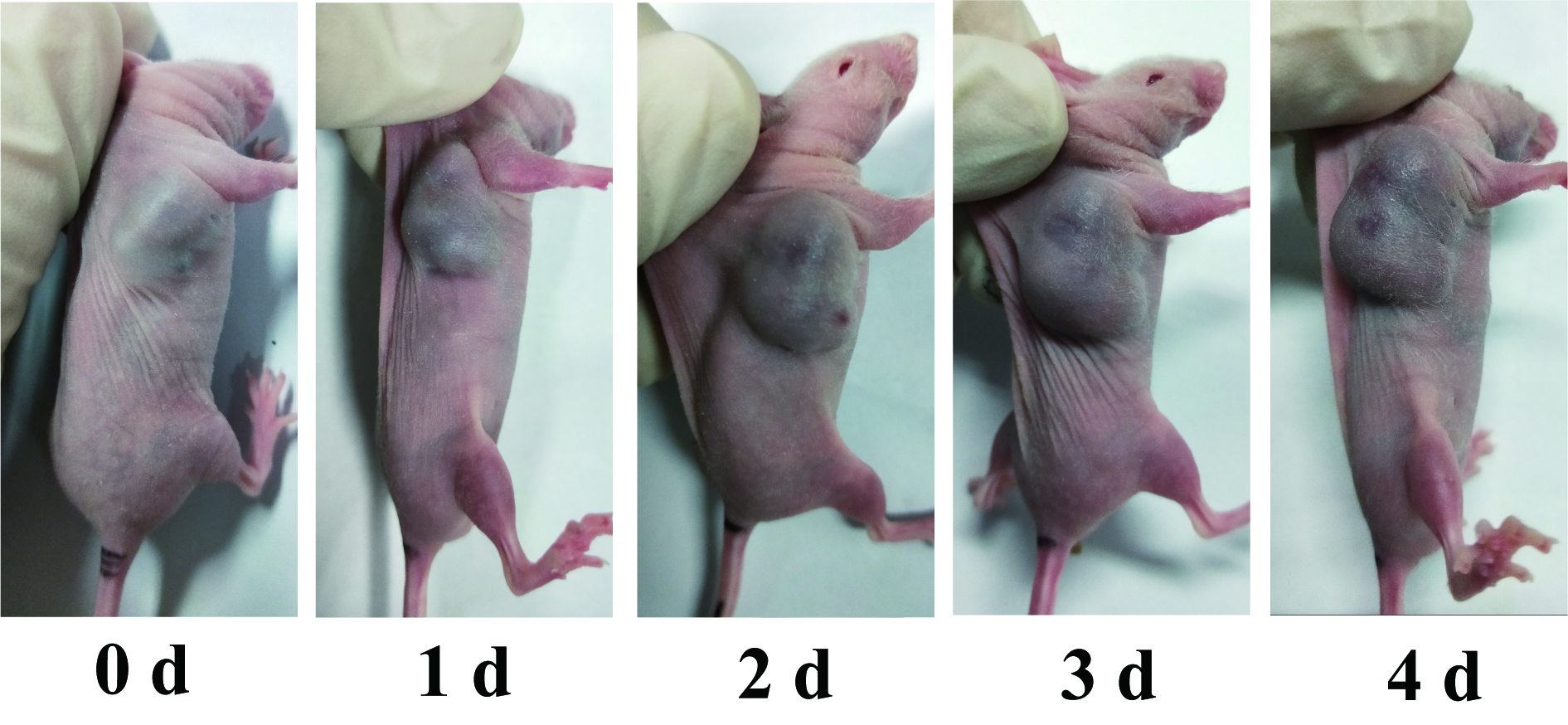

Supplement: Supplementary file 9 — Additional file 9: Fig S9. Photographs of tumor-bearing mice in the control group (untreated). [file 12951_2024_2471_MOESM9_ESM.tif]

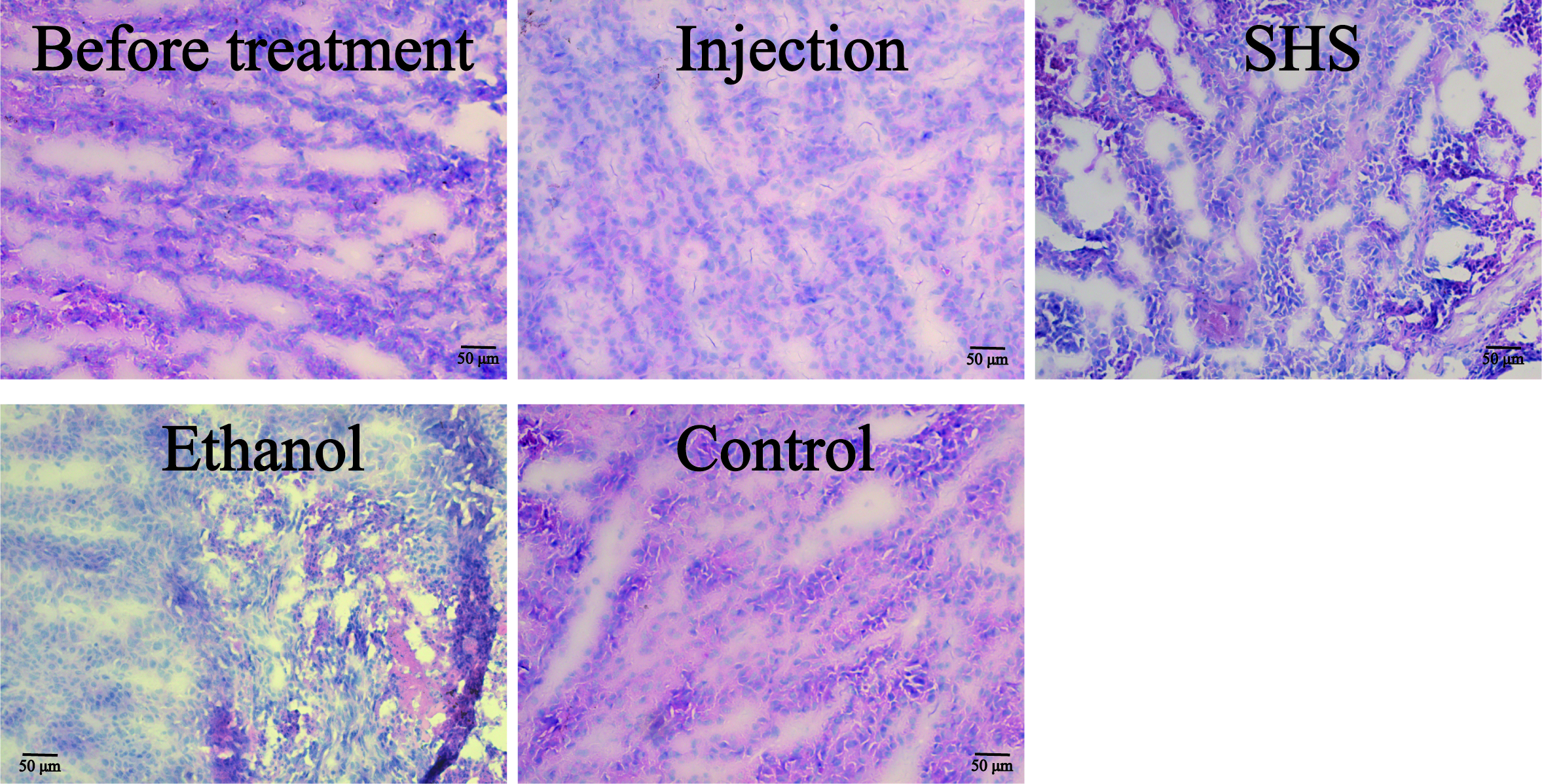

Supplement: Supplementary file 10 — Additional file 10: Fig S10. H&E analysis of B16 tumors dissected from mice before (0 d) and after (15 d) treatment. Scale bar was 50 µm. [file 12951_2024_2471_MOESM10_ESM.tif]
